# Dynamic calibration of low-cost PM_2.5_ sensors using trust-based consensus mechanisms

**DOI:** 10.1038/s41612-025-01145-2

**Published:** 2025-07-05

**Authors:** Sachit Mahajan, Dirk Helbing

**Affiliations:** Computational Social Science, ETH Zurich, Zurich, Switzerland

**Keywords:** Environmental sciences, Mathematics and computing

## Abstract

Low-cost particulate matter (PM) sensors enable high-resolution urban air quality monitoring but face challenges from offsets, scaling mismatches, and drift. We propose an *adaptive* trust-based calibration framework that first corrects systematic errors and then dynamically adjusts model complexity based on sensor reliability. Extensive simulations and real-world deployment in Zurich, Switzerland validate the approach. Each sensor’s trust score integrates four indicators: accuracy, stability, responsiveness, and consensus alignment. High-trust sensors receive minimal correction, preserving baseline accuracy, while low-trust sensors leverage expanded wavelet-based features and deeper models. Results show mean absolute error (MAE) reductions of up to 68% for poorly performing sensors and 35–38% for reliable ones, outperforming conventional calibration methods. By using trust-weighted consensus, the framework reduces dependence on large training datasets and frequent re-calibrations, ensuring scalability. These findings demonstrate that dynamic, trust-driven calibration can substantially enhance low-cost sensor network accuracy across both controlled scenarios and complex real-world environments.

## Introduction

Air quality monitoring in urban environments has become increasingly essential due to the growing recognition of the impact of particulate matter (PM) on public health and environmental quality. The adverse effects of exposure to PM are well-documented, ranging from respiratory to cardiovascular diseases, with especially severe impacts on vulnerable populations^1,2^. Despite the acknowledged importance of air quality data, reference-grade monitoring stations remain sparsely distributed, because of their substantial costs. This often results in significant gaps in the spatial and temporal coverage of pollution measurements^3–5^. These gaps are especially evident in urban settings, where numerous emission sources and complex geometries contribute to fine-scale variability in pollution levels^6^.

The rise of low-cost sensors (LCS) offers a promising avenue to address these coverage limitations. Such sensors, often available at a fraction of the cost of reference-grade monitors, enable dense deployments that capture granular pollution patterns previously undetectable with conventional approaches^5–10^. Rapid advancements in sensor technology have further enhanced LCS accuracy, with some devices approaching reference-grade performance under controlled conditions^11^. Their low cost encourages participatory initiatives, allowing local authorities, citizen scientists, and researchers to deploy extensive sensor networks that enhance existing monitoring systems^12–14^.

Despite these advantages, the deployment of LCS poses substantial challenges, particularly in ensuring consistent data reliability. Low-cost devices remain susceptible to various confounding factors such as humidity-induced particle growth, temperature variations, and the innate limitations of light-scattering techniques^15–17^. These effects can lead to systematic measurement biases that may worsen in heterogeneous urban environments, where diverse aerosol compositions and rapidly shifting meteorological conditions amplify sensor response variability^18,19^.

Traditional calibration approaches often involve co-locating LCS with reference-grade instruments and perform either linear regression or more advanced statistical corrections^20–22^. While effective under controlled scenarios, these methods commonly fail to capture the non-linear dependencies between sensor responses and environmental conditions. More sophisticated machine learning approaches, such as neural networks or random forests, can enhance calibration^17,23,24^, but typically demand large training data sets and considerable computational resources, limiting their scalability. Additionally, the opacity of many machine learning models (often framed as “black box algorithms”) has raised concerns regarding their interpretability and explainability, thereby obstructing regulatory and public acceptance^25,26^. A further concern lies in sensor drift, wherein the performance of LCS deteriorates over time, undermining long-term reliability, unless costly and logistically complex periodic re-calibrations are being performed^27–30^. Thus, despite their promise for high-resolution monitoring, low-cost sensor networks often fall short of fulfilling their potential in operational contexts^31–33^.

In light of these challenges, we explore an approach inspired by democratic voting processes and collective intelligence. Democratic systems often rely on the “wisdom of crowds,” wherein aggregated independent opinions produce more robust outcomes than a single expert judgment^34^. Analogously, participatory sensing systems have shown that blending multiple sensor measurements can yield enhanced accuracy compared to relying on any single sensor alone^35,36^. Transferring these concepts to air quality monitoring, however, is non-trivial. Measurements reflect diverse local conditions, and sensor behaviors can vary drastically due to deployment settings and sensor-specific drift. Consensus-based approaches must, therefore, adapt to spatio-temporal variations, while effectively identifying unreliable readings and reducing their influence^37^. Additionally, LCS often show systematic biases in both, mean and variance, motivating the initial step of offset-scale correction. Correcting these large-scale errors first provides a more stable baseline for subsequent, more nuanced calibration methods.

In addressing these hurdles, we draw upon trust metrics—similar to reputation mechanisms in online platforms—to account for each sensor’s historical behavior and degree of agreement with peers^38,39^. Sensors that consistently produce data aligned with reference standards or with trustworthy peers receive a higher weight in the network’s consensus formation process, while those showing anomalies or drift receive a lower weight. This method extends beyond statistical calibration techniques by offering a dynamic, trust-based mechanism that mitigates the risk of propagating erroneous measurements. Unlike many existing solutions that rely solely on frequent reference-based re-calibrations or uniform weighting, our framework tailors sensor corrections via a trust-based computational approach, matching established consensus-forming strategies that are reminiscent of democratic voting processes.

*Accordingly*, in this paper, we present an *adaptive* calibration framework that offers the following key contributions:

- Trust-Based Sensor Evaluation with Consensus: We integrate a continuous trust assessment with consensus-based correction, reducing the reliance on repeated reference co-location. The consensus mechanism treats sensor data like votes, weighted by each sensor’s trust score in a manner analogous to online reputation systems.
- Adaptive Reliability Assessment: Our framework differentiates between initially well-performing and poorly performing sensors, allocating more extensive calibration strategies—such as deeper model architectures or longer rolling windows—to sensors exhibiting lower trust scores.
- Dynamic Weighting of Sensor Contributions: Sensor measurements are weighted based on historical performance and environmental conditions, which is further refined by trust-based consensus computations. This adaptive approach ensures that valuable data from reliable sensors exert greater influence, while problematic sensors do not result in network-wide biases.
- Computational Feasibility and Real-World Applicability: We demonstrate that our approach can be implemented efficiently, making it suitable for real-time or near-real-time sensor network deployments without excessive computational overhead.

By combining collective intelligence principles with robust trust evaluations, our *adaptive* framework overcomes key limitations of existing calibration solutions for LCS.

## Results

### Simulation Results

To systematically evaluate our proposed hybrid calibration framework under controlled yet realistic conditions, we performed a simulation study with two distinct drift scenarios and three different spatial network layouts. The aim was to emulate common real-world factors that contribute to sensor reading inaccuracies: (1) systematic offsets and scaling errors, (2) sensor network topologies with varying spatial correlations, and (3) two types of drift behaviors representing long-term sensor degradation.

We considered two drift conditions that commonly arise in practice:

- {linear: 1.0, exp: 0.0}: a *purely linear drift*, implying that each sensor’s offset grows (or decays) at a nearly constant rate over time.
- {linear: 0.5, exp: 0.05}: a *combined linear-exponential drift*, where sensors exhibit moderate linear degradation combined with an exponential component. This scenario more closely mimics real sensors whose drift accelerates (or slows) as the sensor ages.

These two conditions capture the breadth of likely real-world aging processes.

To explore performance under varying network deployments, we tested three layouts commonly encountered in real sensor networks:

- Grid: Sensors positioned in a regular two-dimensional grid, implying roughly uniform spacing and distance-based correlations.
- Random: Sensors placed uniformly at random in a bounded area, producing heterogeneous spacing.
- Cluster: Sensors concentrated around one or more cluster centers, simulating scenarios where deployment is denser in certain subregions.

Moreover, we evaluated networks of 4, 9, and 16 sensors, covering small, medium, and relatively larger sensor counts for local-scale monitoring.

Each simulated data set spanned 240 time steps (e.g., hours), with a known reference signal incorporating a daily sinusoidal cycle. Sensor readings were generated by combining

- a baseline sinusoid,
- sensor-specific offsets and scaling factors,
- the specified drift condition,
- distance-dependent noise (to replicate spatial correlation).

For each drift condition, layout, and sensor count, we generated 30 replications, leading to a comprehensive set of scenarios. Six calibration methods were then tested: *Uncalibrated*, *Linear Regression*, *Random Forest*, *Gradient Boosting*, *Support Vector Regression (SVR)*, and our *adaptive* framework. Calibration accuracy was measured via mean absolute error (MAE) relative to the reference signal. To ensure a fair and transparent comparison, all baseline models-Linear Regression, Random Forest, Gradient Boosting, and SVR-were trained using the same input features: the raw sensor reading and hour-of-day (encoded as a numeric variable). No domain-specific feature engineering or temporal smoothing was applied, in order to preserve parity with the inputs used by the hybrid calibration method. For all models, we used the default hyperparameters from the scikit-learn library, except for SVR, where we selected a radial basis function (RBF) kernel with *C* = 1.0 and *ϵ* = 0.1. No manual hyperparameter tuning or grid search was performed, to avoid overfitting and maintain consistency across methods. This setup ensures that performance differences reflect differences in calibration logic rather than differences in tuning complexity.

**Fig. 1:**
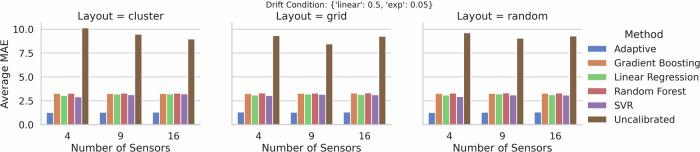
Grouped bar plots of average MAE for two drift conditions {linear: 0.5, exp: 0.05}. The *x*-axis shows the number of sensors; the three panels represent cluster, grid, and random layouts. Note that the uncalibrated data show huge errors in comparison with the calibration methods studied. The new *adaptive* approach performs best.

**Fig. 2:**
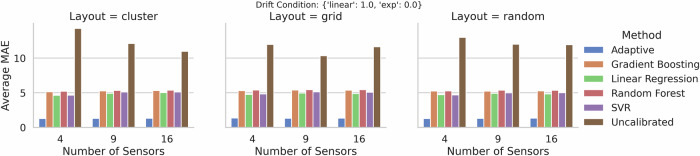
Grouped bar plots of average MAE for two drift conditions {linear: 1.0, exp: 0.0}. Similar to Fig. 1, the *x*-axis represents the number of sensors (4, 9, or 16), while the panels correspond to different layouts of the measurement network (cluster, grid, and random).

Figures 1 and 2 show grouped bar plots of the average MAE for the two drift conditions, respectively. In each figure, the horizontal axis indicates the number of sensors (4, 9, or 16), while the three panels correspond to the **cluster,** **grid**, and **random** layouts. The bar colors represent different calibration methods, including our *adaptive* approach (blue) and the *Uncalibrated* readings (brown).

Our *adaptive* framework performs notably better than established calibration approaches in all tested configurations. The baseline regression-based approaches exhibit moderately high average MAEs, while the *uncalibrated* measurements yield average MAEs that are multiple times higher, in many scenarios exceeding a value of 10. These observations confirm the need for calibration and highlight the advantages of combining offset-scale correction, trust-based modeling, and wavelet-based features, as proposed in our new solution approach.

Table 1 aggregates the MAE across every scenario in our simulation space-i.e., all sensor counts (4, 9, 16), the three layouts (cluster, grid, random), and the two drift conditions. The table reports the mean ± standard deviation of MAE (averaged over 30 replicates). Evidently, the *adaptive* approach yields an overall MAE of 1.31 ± 0.02, which is substantially lower than the errors exhibited by all baseline methods.

**Table 1:** Overall Aggregated MAE across all simulation scenarios (mean ± standard deviation)

Method	Mean MAE	Std MAE
Adaptive	**1.31**	0.02
Gradient Boosting	4.28	1.00
Linear Regression	4.00	0.85
Random Forest	4.33	1.02
SVR	4.02	0.94
Uncalibrated	10.65	1.59

Values in bold indicate the best (lowest) mean MAE.

These findings underscore the robustness of our approach. By simultaneously accounting for sensor-specific offsets, leveraging spatial proximity for consensus, and dynamically adjusting model complexity via trust scores, the *adaptive* method demonstrates superior performance across sensor network sizes, layouts, and drift behaviors. The low standard deviation further indicates that our approach is stable and consistent over repeated trials. This is an important property for real-world deployments, where sensor networks may be subject to unpredictable conditions.

To evaluate the robustness of our calibration framework under abrupt and localized pollution events, we constructed a focused simulation scenario incorporating synthetic PM_2.5_ spikes. These were designed to emulate real-world episodes such as industrial emissions, wildfires, or accidental leakages that can result in sharp, short-term concentration surges.

We generated a 300-hour synthetic time series composed of a smooth sinusoidal background pattern and three distinct pollution spikes with magnitudes of +20, +30, and +25 *μ*g/m^3^, injected at different points in time. To test the framework under heterogeneous sensor behaviors, we created four synthetic sensors: (i) **S1**, which underestimates concentrations and smooths spikes; (ii) **S2**, which overreacts to spikes and introduces random noise; (iii) **S3**, which exhibits a lagged response; and (iv) **S4**, which closely follows the reference with mild scaling errors.

**Fig. 3:**
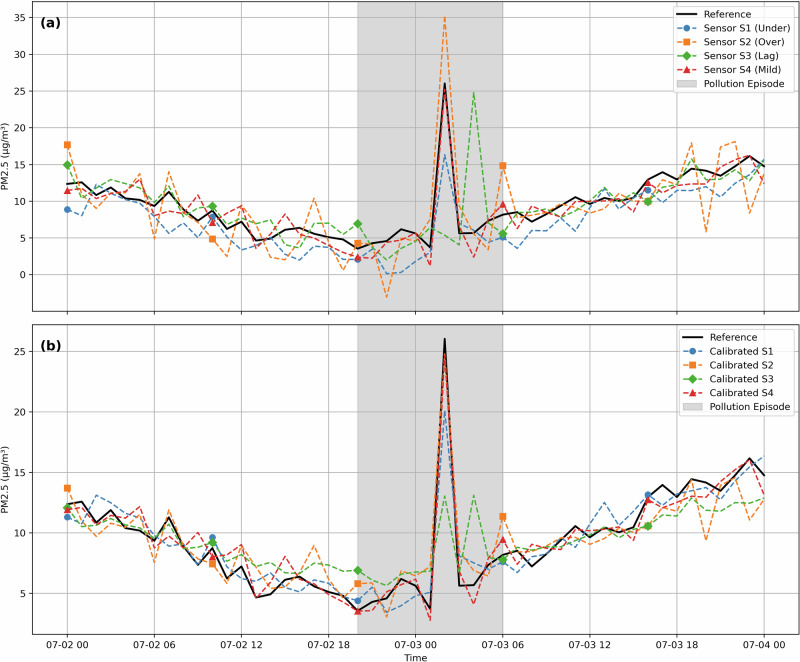
Comparison of PM_2.5_ readings before and after calibration during a simulated extreme pollution event. **a** Raw sensor readings exhibit diverse biases in response to the injected spike. **b** Calibrated outputs using our trust-weighted correction approach align closely with the reference, particularly during the pollution episode. The shaded region highlights the spike event.

For clarity and focus, a 48-hour window was selected from the full simulation to include one prominent pollution event. Figure 3 shows the sensor outputs before (a) and after (b) applying our proposed calibration approach. In subplot (a), we observe the diversity of sensor responses: S1 is delayed and dampened, S2 sharply overshoots the spike, S3 trails behind the true peak, and S4 stays relatively close to the reference. In subplot (b), the calibrated outputs demonstrate clear improvement in aligning with the reference signal, particularly during the spike. The calibration method retains the shape and intensity of the true event for S2, while simultaneously suppressing noise and minimizing overcorrection. For S1, the correction enhances responsiveness to the sharp peak. S4 remains largely unchanged, consistent with its high trust score and already accurate measurements.

Quantitative performance improvements during the event window are reported in Table 2. The proposed method yields substantial reductions in MAE across all sensors: 52% for S1, 50% for S2, 27% for S3, and 35% for S4. These results highlight the framework’s ability to adaptively balance signal fidelity and correction strength, even under volatile and extreme conditions.

**Table 2:** Mean absolute error (MAE) before and after calibration during the visualized pollution episode

Sensor	MAE Before (*μ*g/m^3^)	MAE After (*μ*g/m^3^)
S1	2.35	1.14
S2	2.66	1.32
S3	2.22	1.62
S4	1.33	0.86

### Performance analysis with real-world data

Next, we tested our proposed method on the real-world data. The experimental campaign was conducted at the Empa Dubendorf (suburban) air quality monitoring station in Zurich, where four low-cost sensor units (S1-S4) equipped with Sensirion SPS30 sensors were co-located with a reference-grade PM_2.5_ analyzer. The SPS30 employs a laser-based light-scattering measurement principle to estimate PM_2.5_ concentrations. Airborne particles passing through a laser beam scatter light, and the intensity and angular distribution of this scattered light are used to infer particle size and concentration. This optical approach is known to be sensitive to factors such as humidity, particle composition, and sensor aging-making it particularly suitable for testing our calibration framework, which explicitly accounts for such systematic biases. While our LCS was capable of monitoring multiple parameters, including relative humidity and temperature, this study focuses solely on PM_2.5_ measurements to maintain consistency with the reference station’s available data. Data collection spanned from June 4th to July 21st, 2021, with measurements recorded at hourly intervals, resulting in 1,150 concurrent observations. This summer period captured both stable conditions and pollution episodes, providing a comprehensive data set for evaluating sensor performance across varying concentration levels.

**Fig. 4:**
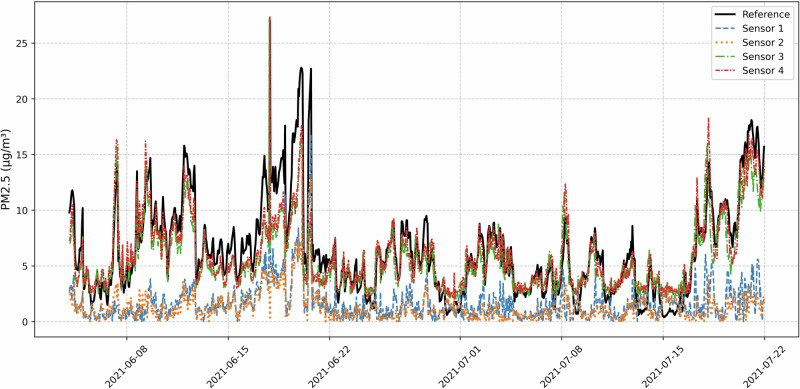
Time series of PM_2.5_ measurements showing the evolution of concentrations throughout the study period. Reference measurements (black solid line) reveal several distinct pollution episodes, particularly in late June and mid-July, with peak concentrations exceeding 20 *μ*g/m^3^. Sensors demonstrate varying response characteristics, with S3 and S4 (green and red lines) closely tracking reference patterns while S1 and S2 (blue and orange lines) show consistent underestimation.

The reference station recorded PM_2.5_ concentrations ranging from 0.4 to 27.0 *μ*g/m^3^, with a mean of 6.42 ± 4.21 *μ*g/m^3^. Figure 4 reveals distinct temporal patterns, including several pollution episodes where concentrations exceeded 15 *μ*g/m^3^. These episodes provide crucial test conditions for evaluating sensor performance under elevated pollution conditions, complementing the more frequent moderate concentration range (2–10 *μ*g/m^3^).

To quantify sensor performance, we employed a comprehensive set of statistical metrics:

- MAE and Root Mean Square Error (RMSE) to assess absolute measurement accuracy
- Pearson correlation coefficient (r) to evaluate temporal pattern reproduction
- Mean bias to identify systematic measurement offsets
- Normalized metrics (NRMSE and Normalized Bias) to enable cross-comparison between concentration regimes

**Fig. 5:**
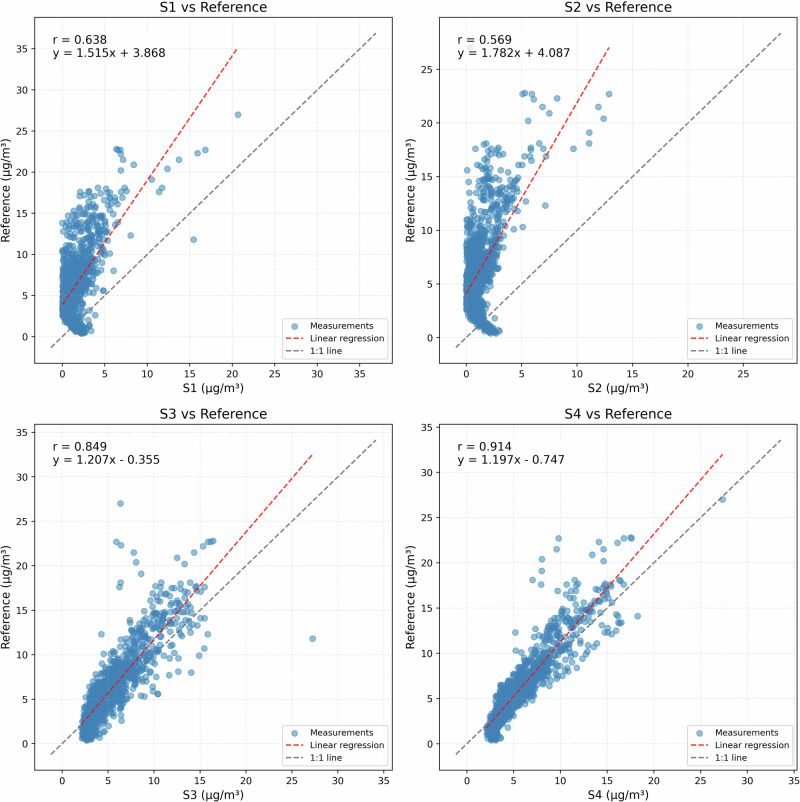
Correlation analysis between low-cost sensors and reference measurements, showing scatter plots with linear regression (red dashed line) and ideal 1:1 response (gray dashed). Regression equations quantify systematic bias (intercept) and sensitivity (slope) for each sensor. S4 demonstrates near-ideal behavior (slope = 1.197, r = 0.914), while S2 shows significant deviation (slope = 1.782, r = 0.569).

Figure 5 reveals distinct performance characteristics across the sensor array. Correlation analysis demonstrates strong linear relationships for S3 and S4 (r = 0.849 and 0.914, respectively), with regression slopes approaching unity (1.207 and 1.197). These sensors exhibit minimal baseline offsets (-0.355 and -0.747 *μ*g/m^3^), indicating good measurement accuracy across the observed concentration range. In contrast, S1 and S2 show moderate correlations (r = 0.638 and 0.569) with substantial deviations from ideal response, evidenced by larger slopes (1.515 and 1.782) and significant positive intercepts (3.868 and 4.087 *μ*g/m^3^). A slope exceeding 1.5 indicates that, for every 1 *μ*g/m^3^ increase in true concentration, the sensor’s reading may rise by around 1.5-1.8 *μ*g/m^3^, thereby exaggerating pollution levels at higher concentrations. In practice, this amplified response can produce increasingly large negative biases, once we subtract the sensor measurement from the true concentration, as reflected in Table 3.

**Table 3:** Performance metrics for each sensor relative to reference measurements

Sensor	MAE (*μ*g/m^3^)	RMSE (*μ*g/m^3^)	Correlation	Bias (*μ*g/m^3^)	NRMSE (%)	Norm. Bias (%)
S1	4.881	5.810	0.638	−4.735	90.5	−73.8
S2	5.239	6.260	0.569	−5.110	97.5	−79.6
S3	1.683	2.444	0.849	−0.807	38.1	−12.6
S4	1.308	1.875	0.914	−0.432	29.2	−6.7

MAE and RMSE quantify absolute accuracy, while normalized metrics enable performance comparison across concentration ranges.

Table 3 confirm these observations quantitatively. S4 demonstrates superior performance across all metrics, with the lowest MAE (1.308 *μ*g/m^3^) and NRMSE (29.2%). S3 shows similar reliability with marginally higher errors. In contrast, S1 and S2 exhibit substantial measurement errors, with MAE exceeding 4.8 *μ*g/m^3^ and normalized biases approaching -80%.

These results highlight three critical insights for low-cost sensor calibration. First, identical sensor models can exhibit markedly different performance characteristics under identical conditions, necessitating individual calibration approaches. Second, the observed combination of varying slopes and intercepts suggests complex response functions that may benefit from non-linear calibration strategies. Third, the strong temporal correlation of even poorly performing sensors indicates potential for improvement through appropriate calibration techniques.

### Error characteristics and dependency analysis

**Fig. 6:**
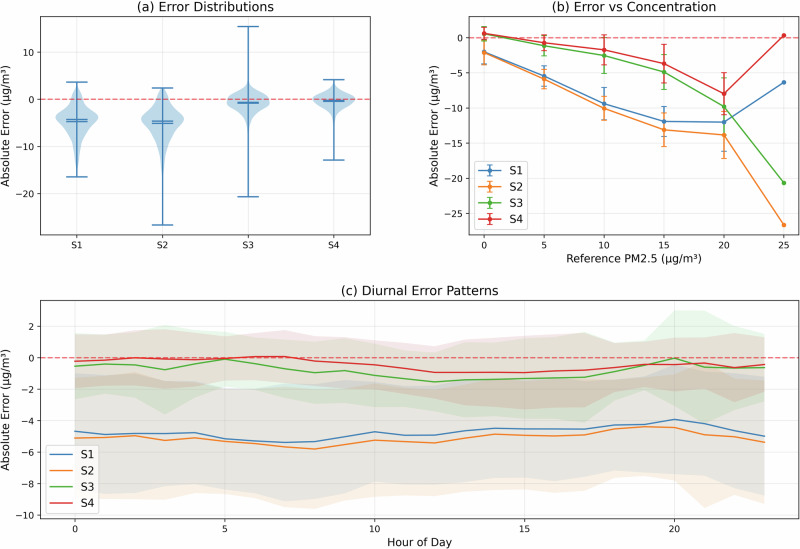
Sensor error characteristics based on raw data. **a** Statistical distributions of measurement errors for each sensor, with violin plots indicating probability densities and horizontal lines marking quartiles. **b** Evolution of absolute errors with increasing PM_2.5_ concentration, demonstrating non-linear relationships and divergent behaviors at higher concentrations. **c** Diurnal patterns in measurement errors, with shaded regions representing one standard deviation uncertainty bands. The red dashed line at zero indicates ideal (error-free) performance in all panels.

To develop an effective calibration strategy, we conducted a comprehensive analysis of measurement errors across different operational conditions. Figure 6 presents three complementary views of error characteristics: their statistical distributions, concentration dependence, and temporal patterns.

The error distributions (Fig. 6a) reveal distinct behavioral patterns among the sensors. S4 demonstrates the most symmetric error distribution centered near zero, with a compact interquartile range indicating consistent performance. S3 shows similar symmetry but with greater variability, particularly in the lower tail of the distribution. In contrast, S1 and S2 exhibit notably asymmetric distributions skewed toward negative errors, reflecting their systematic tendency to underestimate PM_2.5_ concentrations.

An analysis of the error variation with concentration levels (Fig. 6b) uncovers important non-linear relationships. At low concentrations (<5 *μ*g/m^3^), all sensors display relatively small absolute errors within ± 2 *μ*g/m^3^. However, their behaviors diverge significantly as concentrations increase. S4 maintains near-zero mean error across the entire range, with only a modest degradation at higher concentrations. S3 shows a gradual increase in negative bias above 10 *μ*g/m^3^, reaching approximately −20 *μ*g/m^3^ at the highest concentrations. S1 and S2 exhibit the most pronounced concentration dependence, with errors becoming increasingly negative at a rate of approximately −1 *μ*g/m^3^ per 5 *μ*g/m^3^ increase in concentration.

The diurnal analysis (Fig. 6c) reveals subtle but important temporal patterns in sensor performance. While S4 maintains consistent accuracy throughout the day, with mean errors staying within ± 1 *μ*g/m^3^, the other sensors show systematic variations. S3 exhibits slightly larger errors during afternoon hours (10:00–16:00). S1 and S2 maintain relatively stable bias levels throughout the day, but show increased variability (wider uncertainty bands) during early morning hours, possibly related to changing environmental conditions. Such early-morning variability may be tied to stronger temperature inversions or overnight humidity changes, which can differentially affect lower-cost optical sensors. For instance, moisture can cause particle growth or lens fogging, thus altering the light-scattering characteristics. Identifying these time-specific patterns is pivotal to designing calibration models that incorporate diurnal adjustments or humidity correction factors.

These findings have direct implications for the development of calibration strategies. The concentration-dependent error patterns suggest that linear calibration approaches may be insufficient, particularly for S1 and S2. Furthermore, the distinct error characteristics of each sensor support our approach of developing sensor-specific calibration models rather than applying a uniform correction across the entire network.

### Adaptive calibration framework and performance analysis

**Fig. 7:**
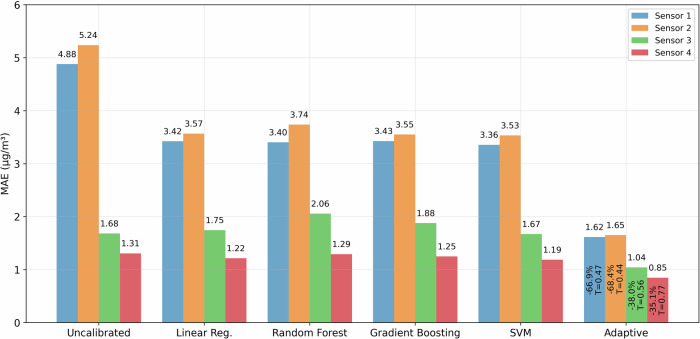
Comparative analysis of calibration methods showing the MAE across different approaches. The *adaptive* approach refers to our two-pass trust-weighted framework, which achieves substantial gains over both uncalibrated measurements and traditional calibration techniques. Sensors with poorer initial accuracy (S1, S2) show the largest reductions in MAE, while even well-performing sensors (S3, S4) benefit from nuanced corrections. Improvements and trust scores (T) highlight how sensor reliability is leveraged in the calibration process.

Our evaluation framework employs a chronological 70–30 temporal split of the dataset, maintaining the sequential nature of measurements to reflect real-world deployment conditions. This temporal separation is crucial for validating the system’s ability to handle evolving environmental factors and potential sensor drift. As shown in Fig. 7, the resulting performance analysis reveals marked improvements across all sensors, with the magnitude of enhancement varying based on each sensor’s initial reliability.

To benchmark the effectiveness of our approach, we evaluated four widely used calibration models: Linear Regression^23,40^, Random Forest^23,41^, Gradient Boosting^40,42^, and SVR^41,43^. These models have been advocated in the literature for low-cost sensor calibration due to their ability to handle non-linear relationships and mitigate measurement noise^40,41,43^. Linear Regression provides a simple baseline that is computationally efficient, whereas Random Forest and Gradient Boosting ensemble methods capture more complex dependencies. SVR employs kernel-based transformations that can learn intricate patterns in sensor data. In comparing these algorithms to our proposed framework, we aim to highlight the value of dynamic trust assessment and adaptive model tuning for sensors with varying reliability.

The framework begins by computing a comprehensive trust score for each sensor, integrating accuracy, stability, responsiveness, and consensus alignment. These trust scores, which in our study range from approximately 0.44 to 0.77, prove highly predictive of each sensor’s baseline performance. For instance, Sensor 4 attains the highest trust value (*T* = 0.77) and exhibits a relatively low uncalibrated error (MAE ≈ 1.31 *μ*g/m^3^), whereas Sensor 2, initially measuring in the 5.24 *μ*g/m^3^ range, receives the lowest trust (*T* = 0.44). Once the trust scores are established, our proposed framework conducts a second pass that leverages a *trust-weighted consensus* feature. In this step, higher-trust sensors collectively exert greater influence on inter-sensor calibration, while less reliable sensors receive correspondingly stronger corrections.

The results in Fig. 7 illustrate how this two-pass design yields clear improvements for each sensor. The well-performing sensors (S3 and S4) achieve a drop in their MAEs by roughly 38% and 35%, respectively, preserving their inherent accuracy while refining it further. More strikingly, however, sensors with weaker initial performance (S1 and S2) achieve MAE reductions of about 67% and 68%, far surpassing the more modest gains of traditional methods like Linear Regression or Random Forest. In some cases, these conventional techniques can even degrade the accuracy of sensors that start off reasonably well. For example, Sensor 3’s uncalibrated MAE is around 1.68 *μ*g/m^3^, yet Random Forest raises this to 2.06 *μ*g/m^3^, illustrating how a standard model may inadvertently penalize an already capable sensor. By contrast, our trust-based approach applies only minimal correction when the trust score is moderately high (0.56 for S3), thereby achieving a further reduction of its MAE to about 1.04 *μ*g/m^3^. This example underscores the importance of adjusting calibration intensity with sensor reliability in order to prevent negative impacts on well-performing sensors.

**Fig. 8:**
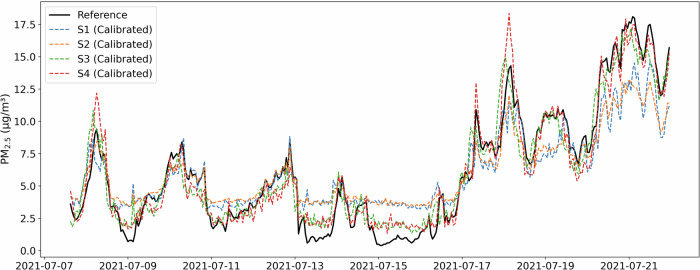
Post-calibration time series for all Zurich sensors, compared to the reference monitor on the test set. After calibration, sensors S1 and S2, which previously showed substantial deviation, now closely track both the temporal variability and absolute levels of the reference station, particularly during pollution peaks and diurnal cycles.

**Fig. 9:**
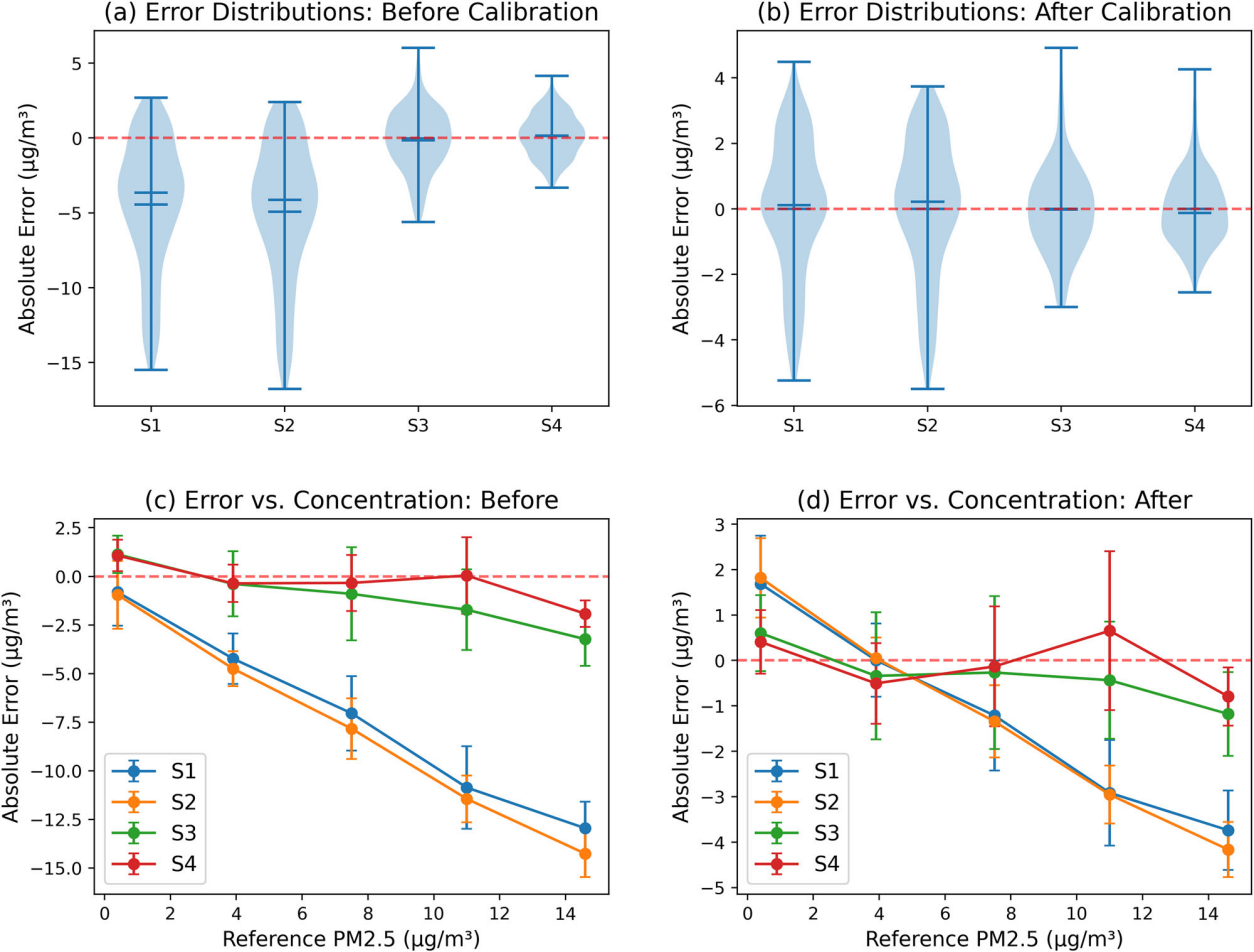
Sensor error characteristics before and after calibration (test set). Top: absolute error distributions for each sensor. Bottom: absolute error as a function of reference PM_2.5_ concentration. Calibration reduces both bias and spread, especially for S1 and S2, confirming effectiveness under real-world conditions.

To visually complement this error-based comparison, Fig. 8 displays the calibrated outputs of all sensors over time on the Zurich test set. The alignment between the corrected sensor readings and the reference signal is clearly visible across the entire period, particularly during sharp transitions and pollution episodes, reinforcing the effectiveness of our adaptive framework. To further visualize the impact of calibration on real-world test data, Fig. 9 presents a direct comparison of error distributions and concentration-dependent behavior before and after applying the proposed framework. Unlike the synthetic or training-based assessments, this comparison is based entirely on the test set, ensuring an unbiased evaluation. The error distributions (top row) show that calibration effectively reduces both bias and variance, particularly for underperforming sensors (S1, S2). Likewise, the bottom panels confirm that the strong underestimation at higher concentrations observed before calibration is substantially corrected. These visual diagnostics complement the quantitative MAE gains reported earlier and emphasize the benefits of trust-weighted adaptation in field deployments.

To further substantiate our findings, we present a comprehensive evaluation of the calibration performance across all models and sensors in Table 4. In addition to the MAE values shown earlier, we report RMSE, Pearson correlation, bias, and NRMSE for each method. This expanded view ensures that the improvements from our framework are not only limited to absolute errors but also preserve structural alignment with the reference signal while minimizing systematic bias.

**Table 4:** Full calibration performance comparison on test set: MAE, RMSE, correlation, bias, and NRMSE. Hybrid consistently improves all indicators across all sensors

Sensor	Method	MAE	RMSE	Corr.	Bias	NRMSE
S1	Linear Regression	3.42	4.29	0.39	0.37	70.28
S1	Random Forest	3.40	4.35	0.41	0.33	71.34
S1	Gradient Boosting	3.43	4.28	0.42	0.42	70.16
S1	SVR	3.36	4.24	0.41	0.22	69.54
S1	**Hybrid**	**1.62**	**2.06**	**0.96**	**0.00**	**33.82**
S2	Linear Regression	3.57	4.59	0.19	0.07	75.22
S2	Random Forest	3.74	4.76	0.16	−0.12	78.01
S2	Gradient Boosting	3.55	4.54	0.23	−0.12	74.49
S2	SVR	3.53	4.55	0.20	−0.14	74.54
S2	**Hybrid**	**1.65**	**2.08**	**0.98**	**0.00**	**34.11**
S3	Linear Regression	1.75	2.28	0.90	1.05	37.46
S3	Random Forest	2.06	2.79	0.86	1.10	45.74
S3	Gradient Boosting	1.88	2.60	0.87	1.02	42.58
S3	SVR	1.67	2.10	0.90	0.70	34.40
S3	**Hybrid**	**1.04**	**1.34**	**0.96**	**0.00**	**21.97**
S4	Linear Regression	1.22	1.64	0.96	0.93	26.94
S4	Random Forest	1.29	1.80	0.94	0.66	29.57
S4	Gradient Boosting	1.25	1.80	0.94	0.64	29.58
S4	SVR	1.19	1.52	0.95	0.47	24.96
S4	**Hybrid**	**0.85**	**1.07**	**0.97**	**0.00**	**17.56**

Bold values indicate the best (most favorable) performance per metric for each sensor.

Our trust-based approach operates on several levels, from considering model complexity (through deeper gradient-boosted trees for low-trust sensors) to integrating trust-weighted consensus values into the feature set. The final blending step combines the raw sensor measurement and the calibrated prediction in proportions determined by each sensor’s trust score. This blend is capped at a maximum weight of 0.8, preventing over-reliance on raw measurements even for high-trust sensors. Altogether, these mechanisms ensure that each sensor benefits from calibration in direct relation to its reliability, avoiding the one-size-fits-all pitfalls of some established methods. Consequently, our approach consistently lowers sensor errors across the network, while preserving the strong performance of sensors that already exhibit good baseline accuracy.

## Discussion

This work presents an *adaptive*, trust-based calibration framework that significantly improves the accuracy of low-cost PM_2.5_ sensors in urban deployments. By coupling a continuous trust scoring mechanism with a consensus- and wavelet-driven calibration scheme, our method tailors correction strategies to each sensor’s reliability and temporal patterns. The experimental results illustrate the impact of this approach: sensors exhibiting initially high errors (S1 and S2) see their MAE reduced by 66.9% and 68.4%, respectively, while even comparatively accurate sensors (S3 and S4) benefit from improvements of 38% and 35.1%. Rather than applying the same calibration adjustments to every sensor, our framework’s trust-based *adaptation* ensures that sensors with poor performance receive more comprehensive corrections, whereas already capable sensors are refined minimally to avoid overshooting corrections, which reduce rather than increase the accuracy.

The improvements we observe are similar to benefits reported in other fields where physics-informed data fusion has been successfully applied. For instance, Treiber et al.^44^ demonstrated that incorporating known traffic propagation characteristics can robustly reconstruct spatiotemporal profiles even with sparse or noisy data. Our approach similarly capitalizes on physical insights to achieve substantial error reductions in sensor calibration.

A key innovation lies in the system’s ability to compute a continuously scaled trust score for each sensor. By evaluating *accuracy*, *stability*, *responsiveness*, and *consensus alignment* over time, the approach captures sensor behaviors that may shift due to drift or environmental factors. The trust score, which in our experiments ranged from about 0.44 to 0.77, governs how much each sensor’s raw reading is trusted relative to its model-generated predictions. For instance, Sensor 4, which attained the highest trust score (0.77), required only modest calibration to improve an already decent MAE. By contrast, Sensor 1’s lower trust score (0.47) triggered a *deeper* adaptive model architecture and a stronger reliance on consensus patterns, driving a major decrease in the error. In a final blending phase, the trust score can allocate up to 80% weight to the raw measurement if reliability is high, mitigating the risk of “over-correction” while still letting poorly performing sensors benefit from more substantial model-based adjustments.

Another essential factor is the initial *offset-scale correction* applied to each sensor’s raw data, which removes large systematic biases upfront. By aligning sensors in both mean and variance before a more nuanced (wavelet-based) calibration is applied, the framework ensures that the subsequent modeling steps can concentrate on residual, less trivial error patterns. The integration of *multi-scale temporal analysis* (via wavelet decomposition) further allows the system to capture short-term fluctuations (e.g., rush-hour spikes) alongside slower pollutant trends. In practice, the wavelet features, when combined with trust-weighted consensus values, effectively tackle abrupt changes while preventing drifts from degrading overall performance. A notable strength is that well-performing sensors are rarely degraded by this second pass, since the trust mechanism confines corrections if baseline accuracy is already strong.

The plausibility and performance of the calibration methods have been explored in simulation studies. Although the empirical dataset used in this study covers a relatively uniform summer period, the robust improvements achieved under these conditions demonstrate the method’s effectiveness. In real-world deployments with more diverse conditions (e.g., seasonal variations or extreme pollution episodes), the *offset-scale* step and the *trust-driven* model should continue to be highly beneficial, as the model can further adapt to a broader range of operating scenarios. In addition, this staged approach remains practical even under limited reference availability, since the trust mechanism and wavelet-based features do not require dense or long historical records to operate effectively. Nevertheless, additional studies using longitudinal datasets and measurements from diverse geographic and meteorological settings would be valuable to further assess the generalizability and robustness of the proposed framework.

Several future directions are possible. One extension involves further enhancing the spatial consensus step by integrating more explicit geo-statistical or meteorological data, enabling the framework to effectively consider macro-level pollution data. Another avenue is refining the model design to account for sensor aging or abrupt failures by dynamically re-calculating offset-scale parameters over time. Moreover, the principles underlying our trust-based adaptive calibration framework may also prove beneficial for other sensor modalities, such as low-cost gas sensors or environmental sensors measuring temperature and humidity, where sensor-specific drift and measurement variability present similar challenges. Overall, our results confirm that a carefully orchestrated combination of a *offset-scale correction*, *continuous trust scoring*, *wavelet-based feature extraction*, and *trust-weighted blending* can significantly enhance network-wide accuracy of low-cost PM sensors. By delivering major error reductions for poorly performing sensors while retaining or further improving accuracy for already capable ones, the proposed *adaptive* method shows strong potential for real-world deployments where sensor reliability and environmental conditions can unpredictably shift.

## Methods

The calibration of low-cost PM sensors presents unique challenges due to their varying reliability and response characteristics. Traditional calibration approaches, which often treat sensors in isolation or apply uniform correction strategies, struggle to address the diverse behaviors of sensors within a network. To address these limitations, we propose a *adaptive* calibration framework that integrates physics-inspired corrections, dynamic trust assessment, and adaptive machine learning. This framework is designed to enhance calibration accuracy and robustness by dynamically adjusting to individual sensor characteristics and leveraging network-wide information. The methodology is organized into four stages: (1) offset–scale correction, (2) dynamic trust assessment, (3) multi-scale feature engineering, and (4) trust-weighted consensus integration. This section details these stages.

### Offset–scale correction

Low-cost PM sensors often show two dominant types of systematic errors: a *mean shift* (constant offset) and a *scaling mismatch* (proportional error). These errors can significantly impact sensor’s accuracy if left uncorrected. The first stage of our framework addresses these errors through a linear transformation derived from reference data.

For a given sensor *s*, let *x*_*s*_(*t*) denote the raw measurement at time *t*, and let *x*_ref_(*t*) denote the corresponding reference measurement. We assume that the relationship between the sensor and reference measurements can be approximated by the following affine transformation:

1 $${x}_{{{\rm{ref}}}}(t)\approx {b}_{s}\left({x}_{s}(t)-{a}_{s}\right),$$

where:

- *a*_*s*_ is the *mean shift*, representing a constant offset in the sensor’s baseline.
- *b*_*s*_ is the *scale factor*, representing a proportional error in the sensor’s variance.

The parameters *a*_*s*_ and *b*_*s*_ are estimated using ordinary least squares (OLS) regression. Specifically, we perform a linear regression of the reference measurements *x*_ref_(*t*) against the raw sensor measurements *x*_*s*_(*t*):

2 $${x}_{{{\rm{ref}}}}(t)=\alpha +\beta {x}_{s}(t),$$

where *α* is the intercept and *β* is the slope. The offset and scale factor are then derived as:

3 $${b}_{s}=\beta ,\quad {a}_{s}=-\frac{\alpha }{\beta }.$$

Using the estimated parameters, the raw sensor measurements are transformed into corrected values ${x}_{s}^{{\prime} }(t)$:

4 $${x}_{s}^{{\prime} }(t)={b}_{s}\left({x}_{s}(t)-{a}_{s}\right).$$

This transformation ensures that the corrected measurements ${x}_{s}^{{\prime} }(t)$ are aligned with the reference measurements *x*_ref_(*t*) in both mean and variance, effectively reducing systematic biases.

### Dynamic trust assessment

After correcting for systematic errors, each sensor *s* is assigned a dynamic trust score *T*_*s*_ ∈ [0.2, 1], which quantifies its reliability based on four performance indicators: accuracy, stability, responsiveness, and consensus alignment. The trust score is used to weight the influence of each sensor in subsequent calibration steps, ensuring that more reliable sensors contribute more significantly to the final output.

The trust score *T*_*s*_ is computed as the average of four normalized indicators, each capturing a distinct aspect of sensor performance:

- Accuracy (*A*_*s*_): Measures how closely the corrected sensor measurements ${x}_{s}^{{\prime} }(t)$ match the reference measurements *x*_ref_(*t*). It is defined as:
  5 $${A}_{s}=\frac{1}{N}\mathop{\sum }_{t = 1}^{N}\exp \left(-\frac{| {x}_{s}^{{\prime} }(t)-{x}_{{{\rm{ref}}}}(t)| }{\epsilon }\right),$$
  where *ϵ* = 2.0 is a sensitivity parameter that controls the penalty for large errors. This metric uses both the individual sensor’s corrected readings and the co-located reference monitor.
- Stability (*S*_*s*_): Quantifies the temporal variability of the corrected sensor measurements. It is computed as:
  6 $${S}_{s}=\frac{1}{N}\mathop{\sum }_{t = 1}^{N}\exp \left(-\frac{{\sigma }_{s}^{\,{\mbox{24h}}\,}(t)}{\overline{{x}_{s}^{{\prime} }}}\right),$$
  where ${\sigma }_{s}^{\,{\mbox{24h}}\,}(t)$ is the 24-hour rolling standard deviation of ${x}_{s}^{{\prime} }(t)$, and $\overline{{x}_{s}^{{\prime} }}$ is the mean of ${x}_{s}^{{\prime} }(t)$ over the same window. This metric uses only the time series from the individual sensor.
- Responsiveness (*R*_*s*_): Evaluates how well the sensor tracks short-term fluctuations in the reference measurements. It is defined as the absolute value of the correlation between the first-order differences of ${x}_{s}^{{\prime} }(t)$ and *x*_ref_ (*t*):
  7 $${R}_{s}=\left\vert {{\rm{corr}}}\left(\Delta {x}_{s}^{{\prime} }(t),\Delta {x}_{{{\rm{ref}}}}(t)\right)\right\vert ,$$
  where $\Delta {x}_{s}^{{\prime} }(t)={x}_{s}^{{\prime} }(t)-{x}_{s}^{{\prime} }(t-\Delta t)$ and Δ*x*_ref_ (*t*) = *x*_ref_ (*t*) − *x*_ref_ (*t* − Δ*t*). This metric compares first-order differences between the individual sensor and the reference monitor.
- Consensus Alignment (*C*_*s*_): Assesses the agreement between the sensor and its neighbors. It is computed as:
  8 $${C}_{s}=\exp \left(-\left\vert {{\rm{corr}}}\left({x}_{s}^{{\prime} }(t),{\bar{x}}_{c}(t)\right)-1\right\vert \right),$$
  where ${\bar{x}}_{c}(t)$ is the spatial consensus, defined as the weighted average of neighboring sensors’ corrected measurements:
  9 $${\bar{x}}_{c}(t)=\left\{\begin{array}{ll}\frac{{\sum }_{j\ne s}\exp \left(-\frac{\parallel {{{\bf{p}}}}_{j}-{{{\bf{p}}}}_{s}\parallel }{D}\right){x}_{j}^{{\prime} }(t)}{{\sum }_{j\ne s}\exp \left(-\frac{\parallel {{{\bf{p}}}}_{j}-{{{\bf{p}}}}_{s}\parallel }{D}\right)},\quad &\,{\mbox{if spatial data available}}\,,\\ \frac{1}{| S\setminus \{s\}| }{\sum }_{j\ne s}{x}_{j}^{{\prime} }(t),\quad &\,{\mbox{otherwise}}\,,\end{array}\right.$$
  Here, **p**_*s*_ and **p**_*j*_ denote the spatial coordinates of sensors *s* and *j*, respectively. The parameter *D* controls the spatial decay rate, determining how quickly the influence of nearby sensors diminishes with distance. When spatial positions are available, the consensus is computed as a weighted average using an exponential decay kernel; otherwise, a simple unweighted average across peer sensors is used. This flexibility ensures robustness in both spatially dense and sparse deployment scenarios.

The trust score *T*_*s*_ is computed as the average of the four performance indicators:

10 $${T}_{s}=\frac{1}{4}\left({A}_{s}+{S}_{s}+{R}_{s}+{C}_{s}\right).$$

To ensure robustness, the trust score is assigned in the range [0.2, 1], preventing sensors from being entirely excluded due to transient errors.

### Multi-scale feature engineering

To capture the complex temporal and spatial dynamics of sensor measurements, we construct a multi-scale feature set **F**_*s*_ for each sensor *s*. These features are designed to encode both local and global patterns in the data, enabling more nuanced calibration.

The feature set **F**_*s*_ is organized into four categories:

- Direct Measurements (**D**_*s*_): Includes the corrected sensor measurements ${x}_{s}^{{\prime} }(t)$, the hour of the day *h*, and the first-order differences $\Delta {x}_{s}^{{\prime} }(t)$:
  11 $${{{\bf{D}}}}_{s}=\left\{{x}_{s}^{{\prime} }(t),h,\Delta {x}_{s}^{{\prime} }(t)\right\}.$$
- Wavelet Features (**W**_*s*_): Extracted via discrete wavelet decomposition using the Daubechies-1 wavelet with 3 decomposition levels. These features capture multi-resolution temporal patterns:
  12 $${{{\bf{W}}}}_{s}=\left\{{\psi }_{i,j}\,| \,i=1,2,3;\,j=1,\ldots ,{2}^{i}\right\},$$
  where *ψ*_*i*,*j*_ denotes the wavelet coefficients at level *i* and position *j*.
- Network Features (**N**_*s*_): Incorporate spatial consensus and correlations with other sensors. The trust-weighted consensus ${\bar{x}}_{c}^{{{\rm{trust}}}}(t)$ is computed as:
  13 $${\bar{x}}_{c}^{{{\rm{trust}}}}(t)=\frac{{\sum }_{j\ne s}{T}_{j}{x}_{j}^{{\prime} }(t)}{{\sum }_{j\ne s}{T}_{j}}.$$
- Environmental Features (**E**_*s*_): Include rolling statistics (mean, standard deviation, minimum, maximum) computed over adaptive windows:
  14 $${w}_{{{\rm{min}}}}=\max \left(1,\lfloor 24\cdot (1-{T}_{s})\rfloor \right).$$

This multi-scale feature set provides a comprehensive representation of sensor behavior, enabling the subsequent calibration model to adapt to both local and global patterns in the data.

### Dynamic model architecture

To account for the varying reliability of sensors, we employ an adaptive Gradient Boosting Machine (GBM) approach with hyperparameters that are dynamically adjusted based on the trust score *T*_*s*_. This ensures that sensors with lower trust scores are calibrated using more complex models, while high-trust sensors rely on simpler, more stable models.

For each sensor *s*, the GBM predicts the calibrated value ${\hat{y}}_{s}(t)$ based on the feature set **F**_*s*_(*t*):

15 $${\hat{y}}_{s}(t)={M}_{s}({{{\bf{F}}}}_{s}(t)),$$

where *M*_*s*_ is the GBM model for sensor *s*.

The hyperparameters of the GBM are adjusted as follows:

- Number of Trees: The number of trees *n*_trees_ increases for sensors with lower trust scores:
  16 $${n}_{{{\rm{trees}}}}=100+100(1-{T}_{s}).$$
  This ensures that more complex models are used for less reliable sensors.
- Learning Rate: The learning rate *η* decreases for sensors with lower trust scores to stabilize training:
  17 $$\eta =0.1\cdot {T}_{s}.$$
- Maximum Tree Depth: The maximum depth of each tree *d*_max_ increases for sensors with lower trust scores:
  18 $${d}_{{{\rm{max}}}}=2+2(1-{T}_{s}).$$

To prevent overfitting, additional regularization techniques are employed:

- Subsampling: Each tree is trained on a random subset of the data, with the subsampling rate set to 0.8 for high-trust sensors and 0.7 for low-trust sensors.
- Minimum Samples per Leaf: The minimum number of samples required to split a leaf node is set to 10 ⋅ *T*_*s*_, ensuring that low-trust sensors use more conservative splitting criteria.

The final calibrated value ${\hat{y}}_{s}(t)$ is obtained by combining the corrected sensor measurement ${x}_{s}^{{\prime} }(t)$ with the GBM prediction *M*_*s*_(**F**_*s*_(*t*)) using a trust-weighted blending approach. In the second pass, the spatial consensus ${\bar{x}}_{c}(t)$ is recomputed using trust-weighted averaging:

19 $${\bar{x}}_{c}^{{{\rm{trust}}}}(t)=\frac{{\sum }_{j\ne s}{T}_{j}{x}_{j}^{{\prime} }(t)}{{\sum }_{j\ne s}{T}_{j}}.$$

This ensures that sensors with higher trust scores contribute more significantly to the consensus.

The final calibrated value ${\hat{y}}_{s}(t)$ is computed as a weighted average of the corrected measurement ${x}_{s}^{{\prime} }(t)$ and the GBM prediction *M*_*s*_(**F**_*s*_(*t*)):

20 $${\hat{y}}_{s}(t)=\omega ({T}_{s})\cdot {x}_{s}^{{\prime} }(t)+\left[1-\omega ({T}_{s})\right]\cdot {M}_{s}({{{\bf{F}}}}_{s}(t)),$$

where the weight *ω*(*T*_*s*_) is given by:

21 $$\omega ({T}_{s})=\min ({T}_{s},0.8).$$

This blending approach ensures that even high-trust sensors are not overly reliant on their raw measurements, while low-trust sensors rely more heavily on the GBM predictions.

In practice, a small number of hyperparameters govern the behavior of the framework and are selected using empirical knowledge rather than formal tuning. These include the rolling window size (set to 24 to reflect diurnal cycles), the trust sensitivity parameter *ϵ* = 2.0 in Eq. (5), and the spatial decay rate *D* in Eq. (9), which reflects average sensor separation in urban deployments. A minimum trust score of 0.2 ensures that poorly performing sensors are not fully discarded. These settings proved robust across both simulation and field experiments, requiring minimal adaptation across scenarios.

### Robust training & validation

To ensure the generalizability of the framework, we employ a robust training and validation strategy that mimics real-world deployment scenarios.

The data set is divided into temporal blocks, with each block representing a contiguous time period. The model is trained on historical blocks and validated on future blocks, ensuring that the calibration framework is evaluated under realistic conditions.

To prevent data leakage, all features are scaled using parameters computed exclusively from the training set:

22 $${\tilde{f}}_{k,i}=\frac{{f}_{i}-{\mu }_{i}({{{\mathcal{W}}}}_{ < k})}{{\sigma }_{i}({{{\mathcal{W}}}}_{ < k})}.$$

Herein, *μ*_*i*_ and *σ*_*i*_ are the mean and standard deviation of feature *f*_*i*_ within historical windows ${{{\mathcal{W}}}}_{ < k}$. Missing values are imputed using the mean of non-missing values within a 24-hour rolling window.

To prevent overfitting, training is halted if the validation loss does not improve for 10 consecutive iterations:

23 $$\frac{{{{\mathcal{L}}}}_{\,{\mbox{val}}\,}^{(i-10)}-{{{\mathcal{L}}}}_{\,{\mbox{val}}\,}^{(i)}}{{{{\mathcal{L}}}}_{\,{\mbox{val}}\,}^{(i-10)}} < \epsilon ,$$

where *ϵ* = 0.01 is the improvement threshold.

## Data Availability

The dataset generated and analyzed during this study is available at https://github.com/sachit27/Trust-Based-Calibration-for-Low-Cost-PM2.5-Sensors

## Code availability

The data analysis for this study was performed using Python. All scripts and code supporting the findings are available at https://github.com/sachit27/Trust-Based-Calibration-for-Low-Cost-PM2.5-Sensors
